# Protective effects of apigenin and myricetin against cisplatin-induced nephrotoxicity in mice

**DOI:** 10.1080/13880209.2016.1275704

**Published:** 2017-01-09

**Authors:** Samar M. Hassan, Marwa M. Khalaf, Sawsan A. Sadek, Amira M. Abo-Youssef

**Affiliations:** a Department of Pharmacology and Toxicology, Faculty of Pharmacy, Fayoum University, Fayoum, Egypt;; b Department of Pharmacology and Toxicology, Faculty of Pharmacy, Beni-Suef University, Beni-Suef, Egypt;; c Department of Pharmacology and Toxicology, Faculty of Medicine, Fayoum University, Fayoum, Egypt

**Keywords:** Antineoplastic, prophylaxis, antioxidant, anti-inflammatory, oxidative stress, inflammation

## Abstract

**Context:** Currently, the outcomes of the use of cisplatin in cancer therapy is limited by nephrotoxicity.

**Objective:** This study aims to investigate the nephroprotective role of apigenin and myricetin against cisplatin-induced nephrotoxicity in mice.

**Materials and methods:** Adult female Wistar Albino mice were divided into eight groups (*n* = 8). Group I served as normal control. Groups II, III and IV received apigenin (3 mg/kg, i.p.), myricetin (3 mg/kg, i.p.) or their combination respectively, for seven days. Group V served as positive control group, received vehicles for seven days and cisplatin (7.5 mg/kg, i.p.) for three days starting at day five. Groups VI, VII and VIII received apigenin, myricetin or their combination, respectively for seven days as well as cisplatin injection for three days starting at day five. by the end of the experimental period, a biochemical study involving, nephrotoxicity markers [serum creatinine (Cr) and blood urea nitrogen (BUN)], apoptotic marker [caspase 3], inflammatory mediators [tumour necrosis factor alpha (TNF-α), interleukin 6 (IL-6), cyclooxygenase I and II (COXI, COXII)] and oxidative stress biomarkers [malondialdehyde (MDA), reduced glutathione (GSH) and catalase] was conducted. In addition, renal histopathological alterations were evaluated.

**Results:** Apigenin, myricetin and their combination significantly reduced blood BUN, serum Cr, caspase-3TNF-α, IL-6, COXI and COXII, MDA levels and significantly increased GSH level and catalase activity parallel to, histopathological improvement in kidney tissues.

**Discussion and conclusion:** Apigenin and myricetin exhibited a protective and promising preventive strategy against cisplatin-induced nephrotoxicity due to their antioxidant and anti-inflammatory effects.

## Introduction

Cisplatin (cis-diamminedichloroplatinum II) is one of the platinum-containing chemotherapeutic agents that are highly effective antineoplastic drug (Burger et al. 2011). It is most commonly used in treatment of tumours such as testicular, bladder, lung, stomach and ovarian cancers (Tayem et al. 2006). Nephrotoxicity is considered the major and specific dose-limiting side effect of cisplatin. Cisplatin is cleared by the kidney by both glomerular filtration and tubular secretion (Yao et al. 2007). The primary cisplatin target in kidney is the proximal tubules, where it accumulates and causes cellular damage (Kawai et al. 2005).

The *in vivo* mechanisms of cisplatin-induced nephrotoxicity are complex and involve oxidative stress, apoptosis, inflammation and fibrogenesis. High concentrations of cisplatin result in necrosis in the cells of the proximal tubules, while lower concentrations result in apoptosis (Arany & Safirstein 2003).

Oxidative stress injury is actively involved in the pathogenesis of cisplatin-induced acute kidney injury. Reactive oxygen species (ROS) directly act on cellular components such as lipids, proteins and DNA to destroy their structure (Humanes et al. 2012). The free radicals destroy the lipid components of the cell membrane by peroxidation and denature proteins leading to enzymatic inactivation and result in mitochondrial dysfunction (Davis et al. 2001). Cisplatin may also inhibit antioxidant enzymes such as superoxide dismutase, glutathione peroxidase and catalase.

Furthermore, cisplatin activates tumour necrosis factor alpha (TNF-α) which induces a series of inflammatory changes that mediate renal injury. Cisplatin coordinates the activation of a large network of chemokines and cytokines in the kidney. Therefore, the use of antioxidants or anti-inflammatory agents may play a very important role in prevention of cisplatin induced nephrotoxicity (Durak et al. 2002).

Apigenin and myricetin are naturally occurring flavonoids that are commonly found in tea, berries, fruits and vegetables (Ramesh & Reeves 2004). They have been demonstrated to have various biological activities such as antioxidant and anti-inflammatory effect (Miean & Mohamed 2001; Mira et al. 2002; Singh et al. 2004).

This investigation seeks to elucidate the possible nephroprotective effects of apigenin and myricetin against cisplatin-induced nephrotoxicity in mice and understand their underlying protective mechanisms.

## Materials and methods

### Experimental animals

All the experimental procedures were conducted using adult female Wistar Albino mice, weighing 10–12 g purchased from The National Research Center, Cairo, Egypt. Animals were housed in a temperature (21 ± 2 °C), humidity (60 ± 5%) and light (12 light/dark cycles) controlled facility with access to food and water *ad libitum.* Mice were left to acclimatize for a period of one week prior to the beginning of the study. All experimental protocols were controlled and approved by the Ethics Committee of Faculty of Pharmacy, Beni-Suef University.

### Drugs and chemicals

Cisplatin was obtained from the market Mylan, France. Cisplatin was injected in a dose of 7.5 mg/kg; i.p. for three consecutive days, this dose was selected according to the study of Sahu et al. (2011).

Apigenin, myricetin and all other chemicals included were purchased from Sigma Aldrich. Apigenin (27 mg/mL) was dissolved in dimethylsulfoxide (DMSO) and was injected in a dose of (3 mg/kg, i.p.). Myricetin (10 mg/mL) was dissolved in ethanol and was injected in a dose of (3 mg/kg, i.p.). These selected doses were previously reported not to produce any detectable toxicity on experimental animals (Min et al. 2007; Silvan & Manoharan 2013).

### Experimental design

After acclimatization to the laboratory conditions, the mice were randomly assigned to eight groups (*n* = 6–8) placed in individual cages according to Amr et al. (2008). Group I served as normal control. Group II, III and IV received apigenin (3 mg/kg, i.p.), myricetin (3 mg/kg, i.p.) or combination of apigenin and myricetin, respectively, for seven consecutive days. Group V received vehicles for seven consecutive days and cisplatin (7.5 mg/kg body weight, i.p.) daily in the last three days and served as positive control. Groups VI, VII and VIII received apigenin, myricetin and their combination respectively for seven consecutive days in addition to cisplatin for the last three days.

### Blood sampling and biochemical analysis

By the end of the treatment period, mice were fasted for 12 h, weighed and blood samples were withdrawn from the retro-orbital venous plexus under light anaesthesia using heparinized microhematocrit capillary tubes and collected in centrifuge tubes. Blood samples were allowed to clot at room temperature, and then serum was separated by centrifugation for 10 min at 5000 rpm. Serum samples were used for the determination of blood urea nitrogen (BUN), creatinine (Cr), malondialdehyde (MDA), reduced glutathione (GSH), TNFα, interleukin-6 (IL-6), cyclooxygenase I (COX I), cyclooxygenase II (COX II), catalase and caspase-3 levels.

Following blood samples collection, animals were euthanized and the whole kidneys were excised and weighed. Kidney tissues were fixed in 10% neutral buffered formalin and embedded into paraffin blocks for histopathological evaluation.

## Determination of the chosen parameters

### Determination of S.Cr and BUN levels

Serum creatinine and urea levels were estimated using commercial kits purchased from BioAssay Systems according to the methods described by Fawcett and Scott (1960) and Bartels et al. (1972), respectively. Results were expressed as mg/dL.

### Determination of caspase-3 activity

Serum caspase-3 was determined using a sandwich enzyme immunoassay kit obtained from Cloud-Clone Corp. according to the method described by Cohen (1997). Results were expressed as ng/mL.

### Determination of serum TNFα and IL-6 levels

Serum TNF-α and IL-6 expressed as pg/mL were estimated using solid phase two-site enzyme immunoassay diagnostic kit (Koma Biotech Inc., Korea) and ELISA kit provided from Immuno-Biological Laboratories Inc., respectively. Procedures were performed according to the methods described by Chan and Perlstein (1987) and Bataille et al. (1989), respectively.

### Determination of serum consumed COX- I and COX-II levels

Inflammatory enzymes including COX-I and COX-II were assayed using ELISA kit, obtained from Glory Science Company (St Del Rio) according to the method of Van Weemen and Schuurs (1971).

### Determination of serum oxidative stress biomarkers

Serum MDA level was determined using reagent kit obtained from Cell Bio-labs Inc. (USA) and expressed as μmol/mL. Determination of serum GSH was done using ELISA reagent kit obtained from ShangHai BlueGene Biotech Co. (China) according to the method described by Beutler et al. (1963) and results were expressed as nmol/mL. Serum catalase was estimated using immunoassay kit obtained from EI Arab Co. (China) according to the method described by Góth (1991) and expressed as U/mL.

### Histopathological examination

Kidneys from mice were dissected out and tissue sections were fixed in 10% phosphate buffered formalin solution at room temperature according to Vickers et al. (2004). After an overnight wash, specimens were dehydrated in graded ethanol, cleared in xylene and paraffin embedded. Paraffin-embedded tissue sections of kidneys (4–5 μm in thickness) were obtained according to the routine procedures, mounted on slides and kept at room temperature. Thereafter, slides were stained with haematoxylin and eosin and evaluated by light microscopy.

### Statistical analysis

All data are expressed as means ± SEM (standard error of the mean). Statistical analysis was done using statistical package for social sciences (SPSS) computer software (version 22). One-way analysis of variance (ANOVA) test was used to elucidate significance among group means, followed by Tukey–Kramer *post hoc* test for multiple comparisons. Differences were considered significant at *p* < 0.05.

## Results

### Effects of apigenin, myricetin or their combination on kidney function

Cisplatin-treated group produced a significant increase in serum creatinine and BUN levels as compared to the normal control group. These effects were pronouncedly alleviated by pretreatment with apigenin, myricetin or their combination (Table 1).

**Table 1.** Effects of apigenin, myricetin or their combination on kidney function tests.

Parameters Drugs	Creatinine level (mg/dL)	BUN level (mg/dL)
Control	0.57 ± 0.016	25.5 ± 1.5
Apigenin	0.56 ± 0.32	25.25 ± 1.3
Myricetin	0.48 ± 0.02	22.5 ± 0.71
Apigenin + Myricetin	0.45 ± 0.03	21.37 ± 0.59
Cisplatin	1.6 ± 0.173*	58 ± 1.15*
Cisplatin + Apigenin	0.83 ± 0.03^a^	41.7 ± 1.1^a^
Cisplatin + Myricetin	0.77 ± 0.025^a^	37.2 ± 2.08^a^
Cisplatin + Apigenin + Myricetin	0.62 ± 0.02^a^	29.6 ± 0.67^a,b,c^

### Effects of apigenin, myricetin or their combination on caspase-3 activity

**Figure 1.**
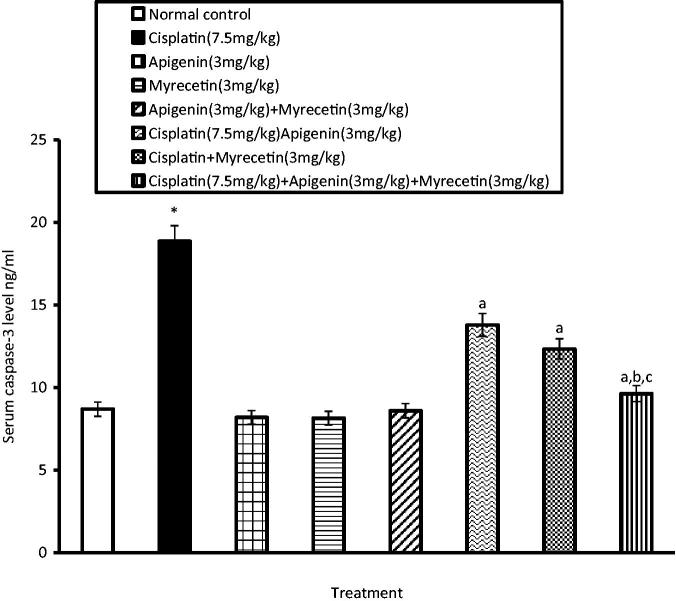
Effects of apigenin, myricetin or their combination on serum caspase-3 activity. Data were expressed as mean ± SEM (*n* = 6–8). *Significantly different from the normal control group at *p* < 0.05. ^a^Significantly different from cisplatin group at *p* < 0.05. ^b^Significantly different from apigenin group at *p* < 0.05. ^c^Significantly different from myricetin group at *p* < 0.05.

Cisplatin-induced nephrotoxicity was associated with increased serum caspase-3 activity indicating apoptosis of renal cells. Pretreatment with apigenin, myricetin or their combination significantly reduced serum caspase-3 activity as compared to cisplatin treated group (Figure 1).

### Effects of apigenin, myricetin or their combination on TNFα, IL-6, COXI and COX II levels

**Figure 2.**
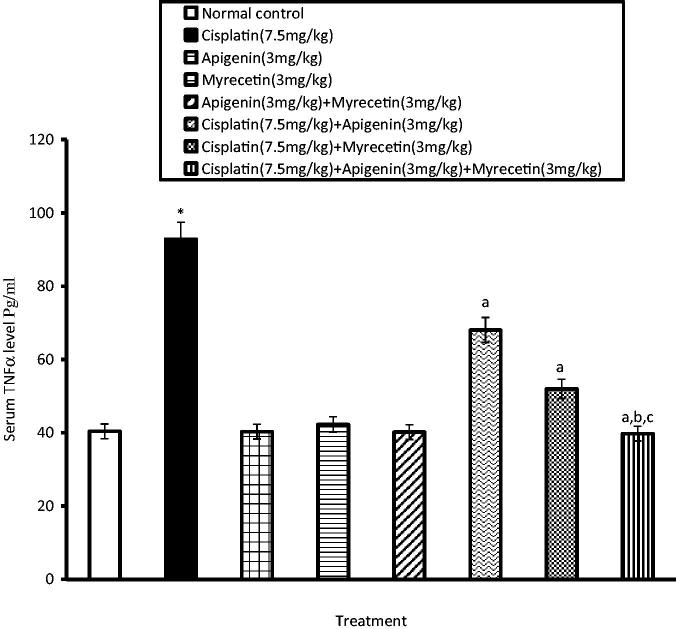
Effects of apigenin, myricetin or their combination on serum TNFα level. Data were expressed as mean ± SEM (*n* = 6–8). *Significantly different from the normal control group at *p* < 0.05. ^a^Significantly different from cisplatin group at *p* < 0.05. ^b^Significantly different from apigenin group at *p* < 0.05. ^c^Significantly different from myricetin group at *p* < 0.05.

**Figure 3.**
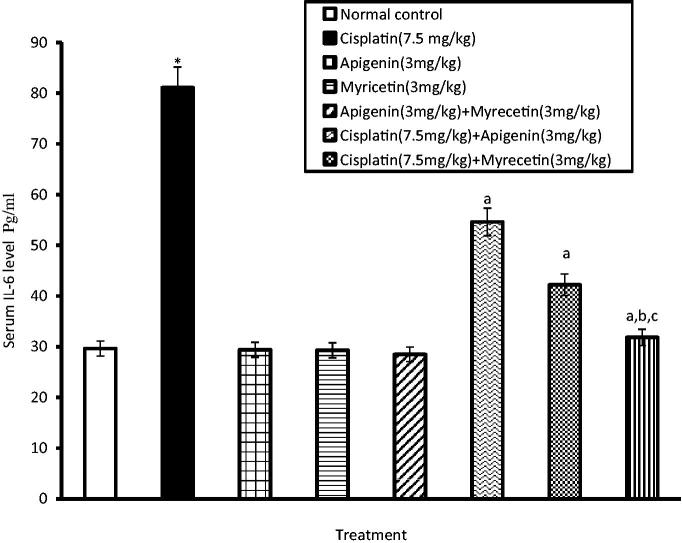
Effects of apigenin, myricetin or their combination on serum IL-6 activity. Data were expressed as mean ± SEM (*n* = 6–8). *Significantly different from the normal control group at *p* < 0.05. ^a^Significantly different from cisplatin group at *p* < 0.05. ^b^Significantly different from apigenin group at *p* < 0.05. ^c^Significantly different from myricetin group at *p* < 0.05.

Treatment of mice with cisplatin revealed a significant increase in serum levels of TNFα and IL-6 compared to normal control group. On the other hand, treatment with apigenin, myricetin or their combination significantly alleviated the aforementioned increase in serum levels of TNFα (Figure 2) and IL-6 (Figure 3).

**Figure 4.**
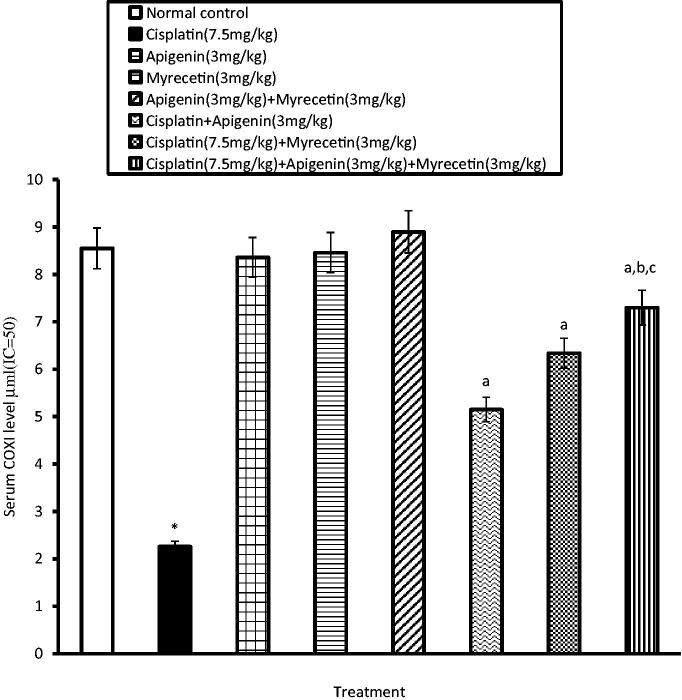
Effects of apigenin, myricetin or their combination on serum COXI level. Data were expressed as mean ± SEM (*n* = 6–8). *Significantly different from the normal control group at *p* < 0.05. ^a^Significantly different from cisplatin group at *p* < 0.05. ^b^Significantly different from apigenin group at *p* < 0.05. ^c^Significantly different from myricetin group at *p* < 0.05.

**Figure 5.**
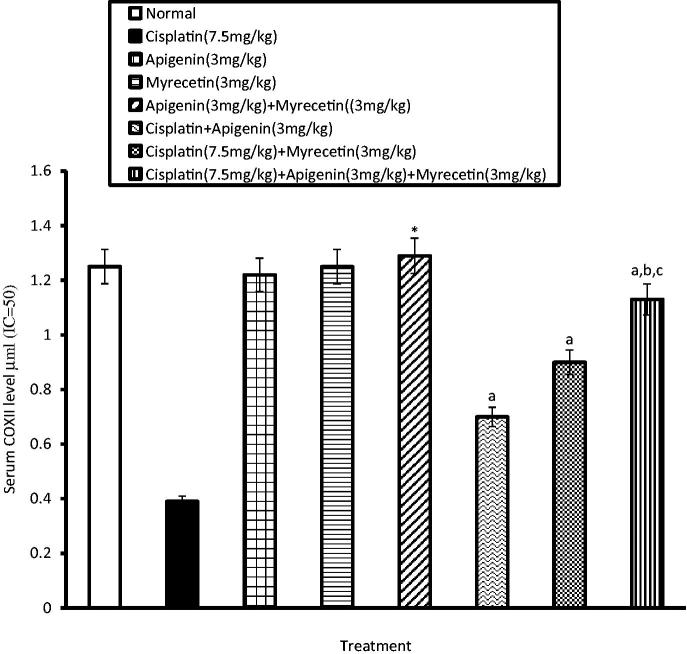
Effects of apigenin, myricetin or their combination on serum COXII level. Data were expressed as mean ± SEM (*n* = 6–8). *Significantly different from the normal control group at *p* < 0.05. ^a^Significantly different from cisplatin group at *p* < 0.05. ^b^Significantly different from apigenin group at *p* < 0.05. ^c^Significantly different from myricetin group at *p* < 0.05.

Similarly, the activity of COXI and COXII in the serum was significantly increased in cisplatin-treated group as evidenced by decrease in the serum level of consumed COXI and COXII, respectively. Co-administration of apigenin, myricetin or their combination with cisplatin significantly decreased serum activity of both COXI (Figure 4) and COXII compared to cisplatin-treated group (Figure 5).

### Effects of apigenin, myricetin or their combination on serum oxidative stress biomarkers

Cisplatin raised serum MDA level compared to the normal control group. It also exhibited a significant decrease in serum GSH level and serum catalase activity as compared to the vehicle control group. Regarding groups pretreated with apigenin, myricetin or their combination, they restored the normal serum levels of MDA, GSH and catalase activity (Table 2).

**Table 2.** Effects of apigenin, myricetin or their combination on serum oxidative stress biomarkers.

Parameters Drugs	Serum GSH Level (μmol/mL)	Serum MDA Level (n mol/mL)	Serum catalase Activity(U/mL)
Control	3.47 ± 0.14	10.48 ± 0.43	15.67 ± 0.309
Apigenin	3.2 ± 0.15	10.53 ± 0.44	15.36 ± 0.34
Myricetin	3.42 ± 0.12	8.92 ± 0.26	15.53 ± 0.37
Apigenin + Myricetin	3.89 ± 0.14	7.22 ± 0.18	16.22 ± 0.32
Cisplatin	0.91 ± 0.037*	25.3 ± 1.51*	4.23 ± 0.232*
Cisplatin + Apigenin	1.7 ± 0.09^a^	17.8 ± 0.37^a^	9.05 ± 0.29^a^
Cisplatin + Myricetin	2.21 ± 0.16^a^	14.3 ± 0.24^a^	10.24 ± 0.39^a^
Cisplatin + Apigenin + Myricetin	2.89 ± 0.78^a^^,^^b^	11.28 ± 0.29^a^^,^^b^^,^^c^	12.42 ± 0.34^a^^,^^b^^,^^c^

Data were expressed as mean ± SEM (*n* = 6–8).

* Significantly different from the normal control group at *p* < 0.05.

a Significantly different from cisplatin group at *p* < 0.05.

b Significantly different from apigenin group at *p* < 0.05.

c Significantly different from myricetin group at *p* < 0.05.

### Effects apigenin, myricetin or their combination on renal histopathological examination

**Figure 6.**
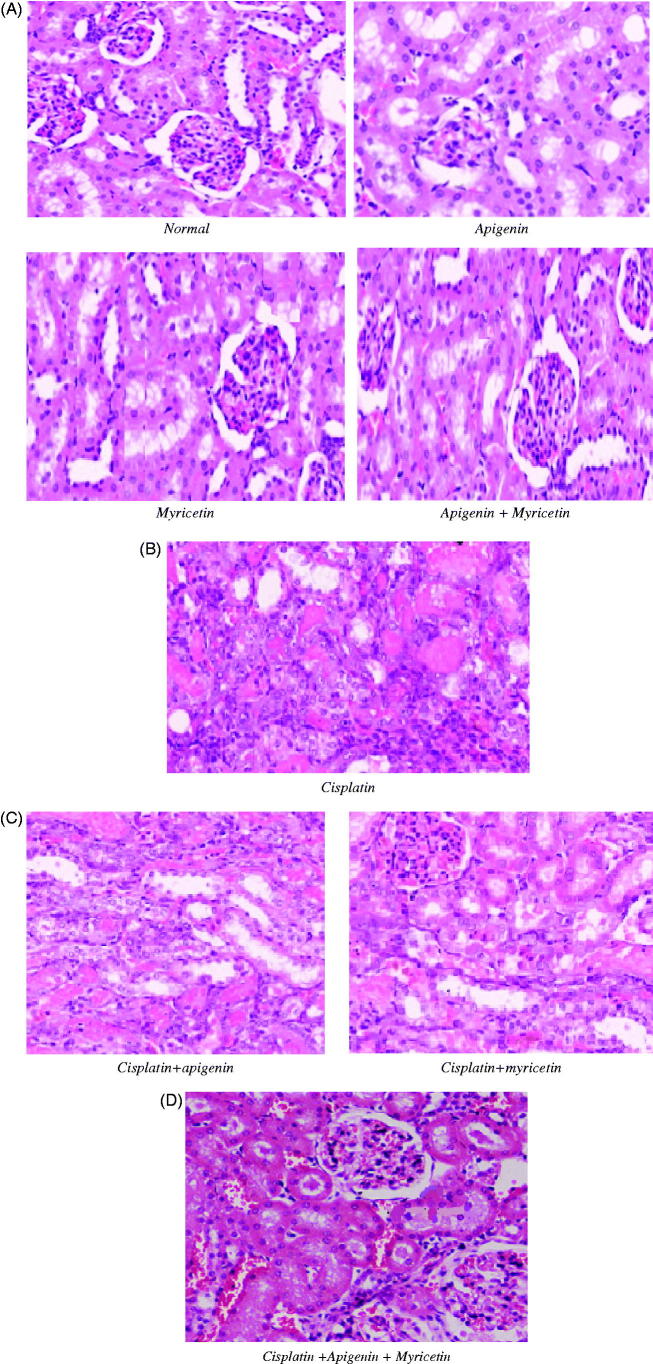
(A) Photomicrographs stained with hematoxylin and eosin from mice kidney sections of normal control, apigenin, myricetin and their combination. These sections showed normal renal histological picture. (B) Photomicrographs stained with hematoxylin and eosin from mice kidney sections of Cisplatin-treated group showing extensive tubular necrosis, tubular dilatation, vacuolization and cast formation. (C) Photomicrographs stained with hematoxylin and eosin from mice kidney sections of Cisplatin-treated groups with apigenin and myricetin displaying remarkable improvement in the histological appearance. (D) Photomicrographs stained with hematoxylin and eosin from mice kidney sections of Cisplatin-treated groups with apigenin and myricetin combination showing nearly normal histological appearance.

Kidney sections from normal control, apigenin, myricetin and combination of apigenin and myricetin-treated mice showed normal glomerulus and tubules with regular morphology (Figure 6(A)). Histological analysis of the kidneys from cisplatin-treated mice showed severe and wide spread of necrosis with dilatation of proximal tubules which lead to loss of tubular architecture, vacuolization, tubular cell desquamation and intraluminal cast formation (Figure 6(B)). Histological analysis of the kidneys from cisplatin-treated mice pretreated with apigenin, myricetin (Figure 6(C)) or their combination showed less histopathological renal changes (Figure 6(D)).

## Discussion

About 25% of commonly used drugs are potentially nephrotoxic and are known as considerable health and economic burden worldwide. Cisplatin is one of these drugs. It is used in the treatment of many organ cancers however; it may lead to acute renal failure by induction of oxidative damage, tubule-interstitial inflammation and apoptosis/necrosis of renal tubular cells (Frezza et al. 2013).

Although many studies have demonstrated the role of several drugs against cisplatin-induced renal toxicity, the mechanism of nephroprotection remains elusive (Miller et al. 2010). So, searching for planning to prevent nephrotoxicity constitutes an active area of investigation.

Previous research has shown the involvement of oxidative stress in the pathogenesis of cisplatin nephrotoxicity (Pabla & Dong 2008). Hence, it is reasonable to suppose that the use of antioxidant defense of renal tissue by exogenous antioxidants having additional properties such as anti-inflammatory and cytoprotective effect should be a strategy to conserve the kidney from the oxidative damage (Pan et al. 2009).

This study showed that administration of cisplatin to mice induced a marked elevation in serum creatinine and blood urea nitrogen (renal function biomarkers), indicating acute renal failure. These results are in agreement with Pan et al. (2015). Treatment of animals with apigenin or myricetin prior to cisplatin injection showed marked decrease in serum levels of creatinine and blood urea nitrogen, indicating improvement of kidney functions. Similarly, combination of apigenin and myricetin prior to cisplatin restored the previous parameters to normal. These results are in accordance with Yao and Wei (2011) and Ozcan et al. (2012) who documented that; treatment with apigenin or myricetin significantly decreased glomerulosclerosis and reduced serum levels of creatinine and BUN.

Caspase and anti-inflammatory should be mentioned first. It was previously reported that ROS generated as by-products of oxidative metabolism frequently cause injury to cellular macromolecules such as DNA, lipids and proteins (Chirino et al. 2008) leading to lipid peroxidation and protein denaturation.

Thiobarbituric acid reactive substance such as MDA is considered as an index of lipid peroxidation. In addition, protein denaturation will subsequently lead to enzymatic inactivation resulting in a decline in the activity of the antioxidant enzymes such as catalase (Rong et al. 2012).

Another marker of balance between antioxidants and free radicals is the depletion of both GSH and protein thiols due to their reaction with reactive oxygen species. Glutathione is as an essential intracellular reducing agent that helps in the maintenance of thiol groups on intracellular proteins and antioxidant molecules in living organisms (Peterson & Cummings 2005).

In this investigation, accumulation of ROS in the kidney was higher in cisplatin-exposed mice than those protected by apigenin or myricetin. Cisplatin significantly increased serum MDA level, while pretreatment with apigenin, myricetin or their combination significantly decreased MDA production. These results are in agreement with Lee and Choi (2008) and Wang et al. (2014) who demonstrated that apigenin and myricetin inhibit lipid peroxidation and tissue damage by preventing the formation of free radicals. Valdameri et al. (2011) also found that, apigenin is able to quench the lipid peroxidation chain and is capable of shielding the membrane from free radicals which cause injuries.

Furthermore, cisplatin treatment resulted in a significant reduction in, the first line of defense, enzymatic antioxidants like catalase. Pretreatment with apigenin, myricetin or their combination produced a significant protection against cisplatin-induced alteration in antioxidant enzymes levels by restoring them to normal. Our results correspond to results of Sahu and Grey (1996) and Yang et al. (2013).

The second line of defense, GSH, protects against cellular injury caused by oxidative stress either by converting the toxic radicals to nontoxic end products or by scavenging free radicals. Cisplatin was found to cause depletion of both GSH and protein thiols (Rodrigues et al., 2011). Sahu and Grey (1996) and Yang et al. (2013) detected that, pretreatment with apigenin or myricetin restored thiols groups and act as pro-oxidants, especially myricetin.

Our observation confirmed that, cisplatin significantly decreased serum level of GSH as compared to the normal control group. Moreover, pretreatment with apigenin, myricetin or their combination significantly increased serum GSH level. This can be attributed to their radical scavenging ability.

These observations support the hypothesis that cisplatin nephrotoxicity is related to free radical generation and the nephroprotection offered by apigenin or myricetin is due to their antioxidant defense system. In agreement with our hypothesis Patel et al. (2007) and Ding et al. (2012) confirmed that, apigenin and myricetin could effectively remove a variety of ROS.

Kaushal et al. (2001) declared that apoptosis is an important mode of cell death in normal and pathologic states. Caspase-1, -8, and -9 are initiator caspase that activate caspase-3, which is the principal executioner caspase in renal tubules apoptosis.

Our observations add further evidence for the previous reports (Shimmyo et al. 2008; Choi & Kim 2009) that caspase-mediated apoptosis plays a crucial role in the execution of apoptotic cell death in case of cisplatin-induced nephrotoxicity. Prophylaxis of animals with apigenin, myricetin or their combination significantly attenuated apoptotic changes and serum caspase-3 level as compared to cisplatin group.

According to the results obtained from our research we realized that, combination of apigenin and myricetin exhibits better results than those obtained from the single drugs. The level of ROS accumulation in the combination therapy is more significantly diminished than either of the single drugs. This leads to less malondialdehyde formation, less lipid peroxidation, better restoring of thiol groups, increased serum GSH, decreased effect on antioxidant enzymes. Therefore, combination therapy exhibits better nephroprotection than the single drugs.

Cytokines play important roles in the normal physiology of cells. They are related to the immune response, inflammation and tissue injury or repair. Cisplatin activated inflammatory cells and subsequently magnified the inflammatory response by releasing various cytokines (TNF-α and IL-6) leading to renal injury (Ramesh & Reeves 2004).

Funakoshi-Tago et al. (2011) found that apigenin significantly inhibited TNF-α induced nuclear factor kappa B (NF-κB) transcriptional activation. Furthermore, myricetin was reported to execute the inhibitory function for the production of inflammatory cytokines (Lee & Choi 2010).

In this study, pretreatment of mice with apigenin, myricetin or their combination significantly reduced serum levels of TNF-α and IL-6. Therefore, it may be suggested that apigenin and myricetin could alleviate renal injury caused by cisplatin through suppression of the inflammatory response.

Another indication of inflammation is COX enzymes. They catalyze a key step in the conversion of arachidonate to prostaglandin (PGs). Prostaglandins play critical part in numerous biologic processes, involving the regulation of immune function and kidney development (Dubois et al. 1998). In agreement with our results, Bauer et al. (2000) demonstrated that COX was overexpressed in cisplatin treatment due to its induction of pro-inflammatory cytokines which trigger prostaglandins formation.

Pretreatment of mice with apigenin, myricetin or their combination alleviates the decrease in serum levels of consumed COXI and COXII induced by cisplatin. These results are consistent with those of Gutiérrez-Venegas et al. (2013) and Wang et al. (2014) who reported that, apigenin and myricetin are considered to be cyclooxygenase blocker flavonoids that exert potent anti-inflammatory effects against oedema and other inflammatory processes. Combination of apigenin and myricetin exhibited better anti-inflammatory effect than the single drugs through restoring normal serum levels of TNFα, IL-6 and COX.

Histopathological examination of the kidneys obtained from control, apigenin, myricetin or the combination groups implied intact renal architecture with normal glomerulus and tubules. Cisplatin intoxication resulted in loss of architecture, degenerated tubular structures with vacuolization, severe renal damage and severe atrophy of glomerulus evidenced by reduction in its size. The changes obtained in this study run parallel with the changes documented by An et al. (2009) who demonstrated histological changes of mice kidney after cisplatin treatment resulting in acute tubular necrosis which affirms irreversible injury to kidney.

Pretreatment with apigenin or myricetin could not prevent cisplatin nephrotoxicity completely with many degenerating tubules. Combination therapy resulted in excellent protection against cisplatin-induced nephrotoxicity and showed predominant normal kidney morphology.

This histopathological examination performed on kidneys is in full agreement with our biochemical results, as it confirmed the severe renal damage caused by cisplatin and the nephroprotective effect apigenin, myricetin or their combination against cisplatin-induced kidney injury.

## Conclusions

Results of this study clearly indicated that oxidative stress, inflammation and apoptosis/necrosis play a critical role in pathogenesis of cisplatin nephrotoxicity. Pretreatment with apigenin, myricetin or their combination significantly attenuated cisplatin-induced functional and histological renal deterioration. They suppressed the renal oxidative stress with the subsequent lipid peroxidation; constrict generation of pro-inflammatory cytokines and DNA damage (apoptosis/necrosis) as well. Finally, according to the findings of this study, it could be stated that there is additive effect observed upon combining apigenin with myricetin.
